# Extra-Appendiceal Neuroendocrine Expressing Goblet Cell Adenocarcinoma of the Cecum—A Case Report and Brief Review of the Literature

**DOI:** 10.3390/reports8010001

**Published:** 2024-12-26

**Authors:** Alexandra Dinu, Mariana Aşchie, Gabriela Isabela Bălţătescu, Manuela Enciu, Ionuţ Burlacu

**Affiliations:** 1 Clinical Service of Pathology, “Sf. Apostol Andrei” Emergency County Hospital, 900591 Constanţa, Romania; amaiorean@yahoo.co.uk (A.D.); aschiemariana@yahoo.com (M.A.); gabriela.baltatescu@univ-ovidius.ro (G.I.B.); burlacuionut82@yahoo.com (I.B.); 2 Institute of Doctoral Studies, Doctoral School of Medicine, “Ovidius” University of Constanţa, 900573 Constanţa, Romania; 3 Faculty of Medicine, ”Ovidius” University of Constanţa, 900591 Constanţa, Romania; 4 Department of Anatomy, Academy of Medical Sciences of Romania, 030171 Bucharest, Romania; 5 Center for Research and Development of the Morphological and Genetic Studies of Malignant Pathology—CEDMOG, “Ovidius” University of Constanţa, 900591 Constanţa, Romania

**Keywords:** anaemia, cecum, extra-appendiceal, goblet cell adenocarcinoma, immunohistochemistry, neuroendocrine

## Abstract

**Background and Clinical Significance**: Neuroendocrine expressing goblet cell adenocarcinomas (GCAs) are uncommon clinically aggressive tumours of the digestive system, originating almost exclusively in the ileocecal appendix. GCA’s singularity comes from its amphicrine nature, expressing both neuroendocrine and exocrine characteristics. The case report’s objective is to raise awareness of this neoplasia’s possible extra-appendiceal localisation by showcasing a GCA involving the cecum with no detectable appendiceal tumour. **Case Presentation**: The authors present a case of GCA with neuroendocrine expression in an 82-year-old male patient with severe anaemia and comorbidities who underwent a right colectomy and had no histopathological evidence of appendiceal tumour involvement. Immunohistochemical testing was performed using synaptophysin, chromogranin A, neuronal specific enolase, CD56, CDX-2, CK20, CEA, MUC2 and Ki67, thus establishing the final diagnosis of high-grade extra-appendiceal goblet-cell adenocarcinoma of the cecum, G3. The patient died on postoperative day 26 due to pneumonia and acute renal failure in a chronic renal disease context. **Conclusions**: Extremely few cases of extra-appendiceal GCA have been reported. Appendiceal evaluation with the exclusion of this possible origin should be mandatory in such cases for a correct classification. These tumours do not benefit from any official management protocols concerning clinical evaluation, and their treatment is commonly based on the tumour’s stage, as in classical adenocarcinoma.

## 1. Introduction and Clinical Significance

Neuroendocrine-expressing goblet cell adenocarcinomas (GCA) are rare tumours of the digestive tract, most frequently involving the appendix and rarely other colonic segments [1].

Goblet cell adenocarcinoma, formerly known as goblet cell carcinoid, is an aggressive epithelial–neuroendocrine hybrid tumour, accounting for less than 5% of primary appendiceal neoplasms [2,3,4,5,6,7]. Its origin is believed to be the pluripotent stem cells found in the intestinal epithelial crypt base, thus explaining the GCA’s clinicopathological uniqueness of exhibiting both neuroendocrine and exocrine features, with a mixed composition of secretory cells like endocrine cells, goblet cells and Paneth cells [8,9].

GCA is included exclusively in the fifth WHO classification of appendiceal tumours [6]. Few cases of extra-appendiceal GCA were reported in the literature, but their number might be underestimated because of the past belief that GCA can only be found in the appendix [1,3,6,10,11]. Extra-appendiceal GCAs were reported to involve gastrointestinal sites such as the stomach, duodenum, small intestine, colon and rectum [2]. Most extra-appendiceal GCA reports have not conclusively excluded the appendix as a source of the tumour, but appendiceal status review is surely to become mandatory diagnostic criteria in such cases [2].

There are currently no well-established therapeutic guidelines for extra-appendiceal GCA; it is recommended that its management and follow-up be based on the tumour’s stage following the protocol for conventional adenocarcinomas [2,7,8]. The extreme rarity of this tumour makes extensive studies difficult, although specific guidelines would be preferred [1,3,6,10,11].

## 2. Case Presentation

### 2.1. Clinical Presentation

An 82-year-old male patient, known with hiatal hernia, benign prostate hyperplasia, gout, chronic renal disease and chronic heart failure, is admitted to the emergency service of the Clinical Emergency County Hospital “St. Apostle Andrei” Constanţa due to fatigue, headache and vertigo. The patient declared that the symptoms had started two weeks prior to his hospitalisation and gradually worsened.

On admission, the patient showed altered general condition with dehydration signs; physical examination showed pale, dry skin and mucous membranes; vital signs included hypotension (85/59 mmHg), tachycardia (112 beats per minute), tachypnea (30 breaths per minute) and a 37.7 °C (99.86 °F) body temperature.

The laboratory tests demonstrated hypochromic microcytic anaemia due to iron deficiency. The patient also had a low red blood cell (RBC) count, suggesting minimal but chronic blood loss. High serum creatinine and urea levels highlighted a possible renal impairment. Urinalysis, on the other hand, did not show signs of renal degradation (normal density, normal urobilinogen levels and absent protein, glucose, ketone bodies and erythrocytes). Serum glucose and liver enzymes were within normal limits, which, in correlation with the absence of urinalysis abnormalities, suggested a normal hepatopancreatic function. Coagulation tests were within normal values. Inflammatory markers consisted of a normal erythrocyte sedimentation rate (ESR) and high C-reactive protein (CRP). The alkaline reserve was low upon admission, suggesting the existence of metabolic acidosis. Carcinoembryonic antigen (CEA) and prostate-specific antigen (PSA), the tumour markers for the most common types of cancer in male patients (digestive and prostatic), were within normal limits (Table 1).

**Figure 1:**
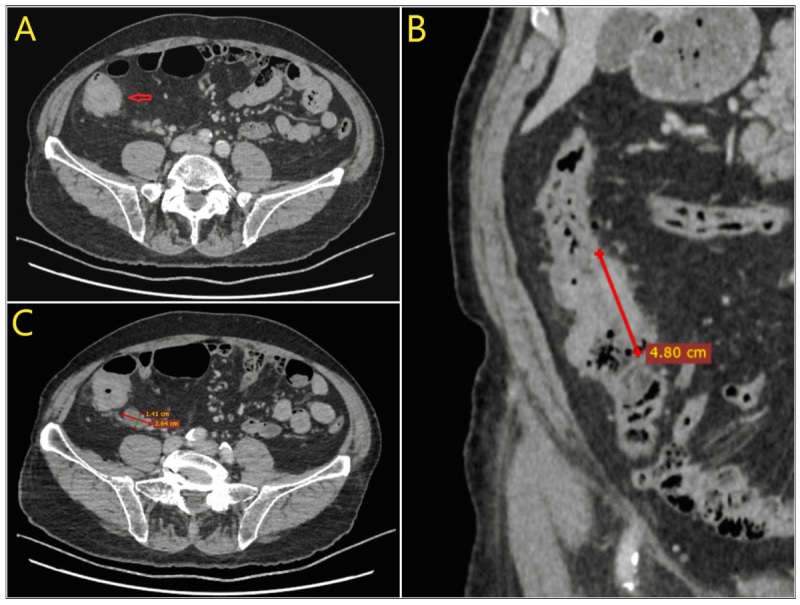
Abdominal CT showed an irregular iodophilic circumferential thickening of the ascending colon wall ((**A**)—arrow), measuring 4.8 cm in length (**B**), which associated perilesional adenopathy (**C**).

Abdominal computed tomography (CT) showed features suggestive of malignancy: an irregular iodophilic circumferential thickening of the ascending colon wall, measuring 4.8 cm in length, with a maximum axial diameter of 1.9 cm, which associated small perilesional adenopathy (Figure 1).

Colonoscopy was recommended and performed, and the biopsy of the cecum tumour was reported as invasive high-grade conventional adenocarcinoma/poorly differentiated G3, with signet ring cells component, with the recommendation that the final histological type of assessment should be performed on the full tumour volume.

**Figure 2:**
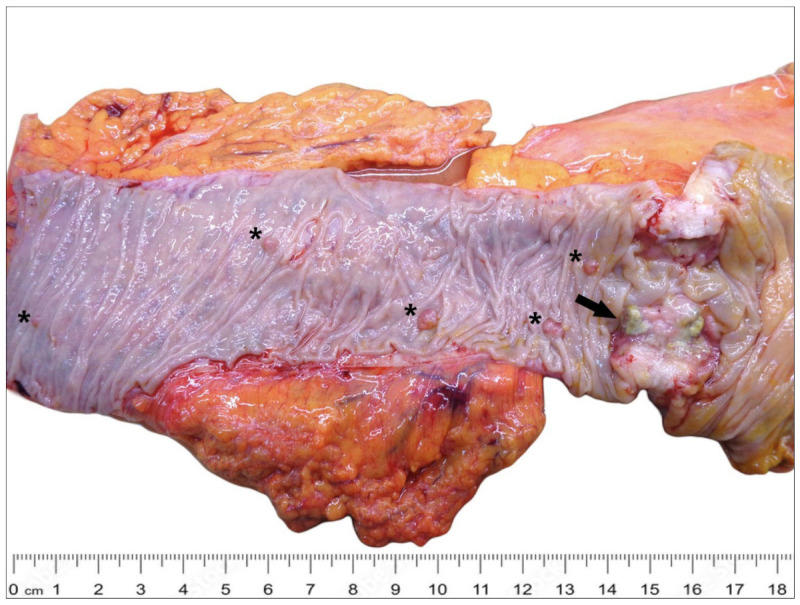
Gross findings showed a 4.5 × 2.5 cm ulcerated exophytic tumour of the cecum producing stenosis (arrow); colonic polyposis was present (asterisk).

The patient underwent a right hemicolectomy with lymph node resection after being transfused. Gross examination revealed a 4.5 × 2.5 cm ulcerated exophytic tumour of the cecum, producing stenosis; colonic polyposis was present (Figure 2).

### 2.2. Pathological Findings

**Figure 3:**
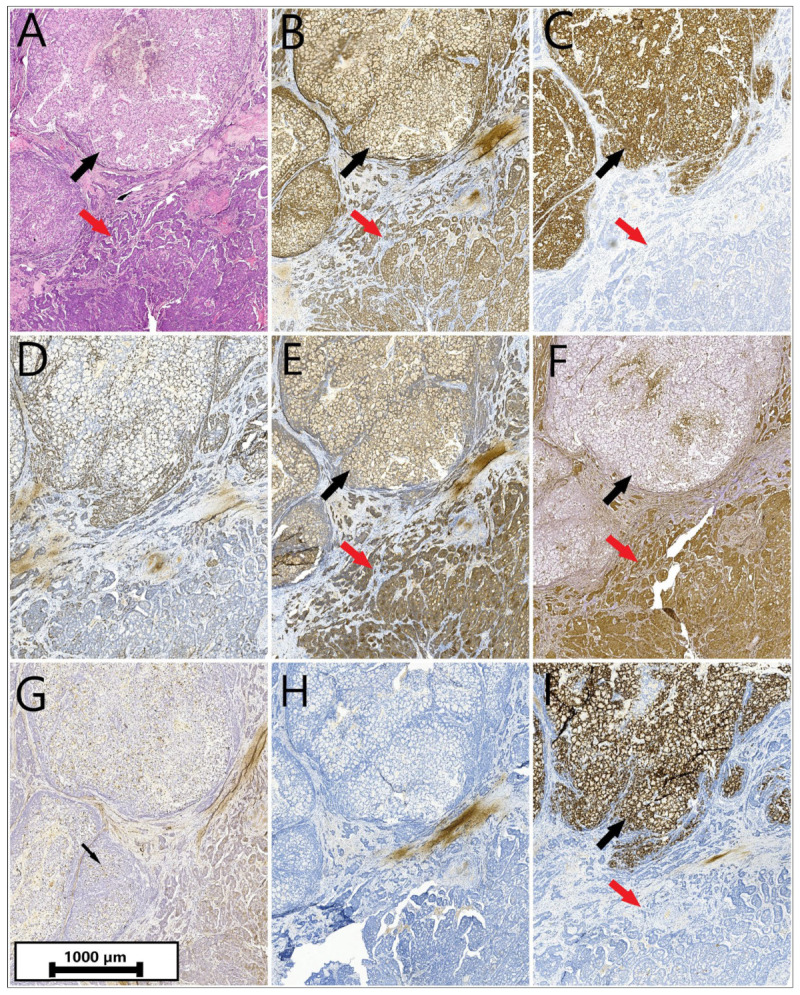
(**A**). Microscopic examination demonstrated goblet-like mucinous cells disposed of in variable-sized clusters in an organoid fashion with signet-ring-like cells (black arrow) and non-mucinous cells with important nuclear atypia, arranged cribriform, in anastomosing tubules and solid sheets (red arrow). Infiltrating small cellular groups/isolated single cells with desmoplastic reaction were present—Hematoxylin and eosin, magnification ×5; (**B**). Immunohistochemistry demonstrating strong and diffuse staining of both mucinous (black arrow) and non-mucinous cells (red arrow) with CDX2—Immunohistochemistry, magnification ×40; (**C**). CK20 intense positive in mucinous cells (black arrow) and negative in non-mucinous cells (red arrow)—Immunohistochemistry, magnification ×40; (**D**). Ki67 staining showed a proliferative pattern of 60%—Immunohistochemistry, magnification ×40; (**E**). Strong and diffuse staining of mucinous (black arrow) and non-mucinous cells (red arrow) with synaptophysin—Immunohistochemistry, magnification ×40. (**F**). Diffuse staining of mucinous (black arrow) and non-mucinous cells (red arrow) with neuronal specific enolase—Immunohistochemistry, magnification ×40; (**G**). Negative CD56—positive control in T cells (black arrow)—Immunohistochemistry, magnification ×40; (**H**). Negative chromogranin A—Immunohistochemistry, magnification ×40; (**I**). Strong staining of the mucinous cells with MUC2 (black arrow); negative reaction in non-mucinous cells (red arrow)—Immunohistochemistry, magnification ×40.

Microscopically, the neoplasm demonstrated goblet-like mucinous cells disposed of in variable-sized clusters in an organoid fashion, accompanied by signet-ring-like cells, and non-mucinous cells with important nuclear atypia, arranged cribriform, in anastomosing tubules and solid sheets (Figure 3A). Infiltrating small cellular groups/isolated single cells with desmoplastic reactions were present. Immunostains were further performed (Table 2).

Neuroendocrine cells were positive for synaptophysin and neuronal specific enolase and negative for chromogranin A; CD56 was negative; CDX-2, CK20 and CEA were intensely positive; goblet cells were immunoreactive for MUC2; Ki67 staining expressed a proliferative pattern of 60% (Figure 3B–I).

**Figure 4:**
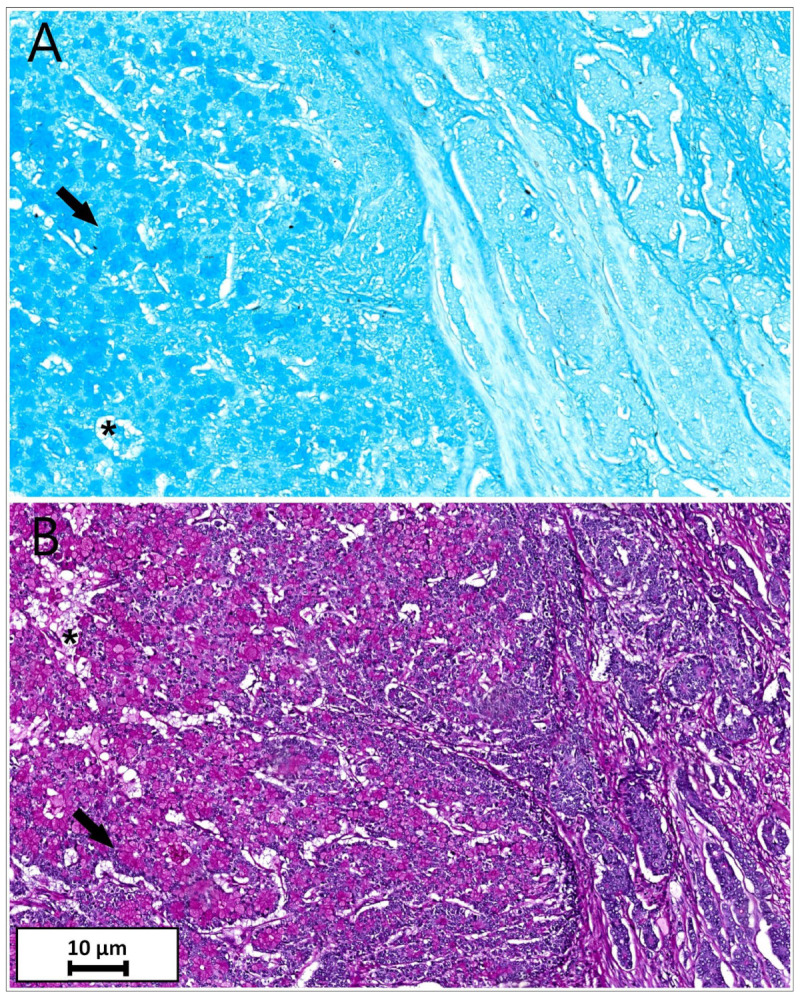
(**A**). Alcian blue special stain demonstrated mucinous cells (arrow) and extracellular mucin (asterisk)—Alcian Blue, magnification ×200; (**B**). PAS blue special stain demonstrated mucinous cells (arrow) and extracellular mucin (asterisk)—PAS, magnification ×200.

PAS and Alcian Blue stains highlighted goblet cells and extracellular mucin with single cells floating within them (Figure 4).

**Figure 5:**
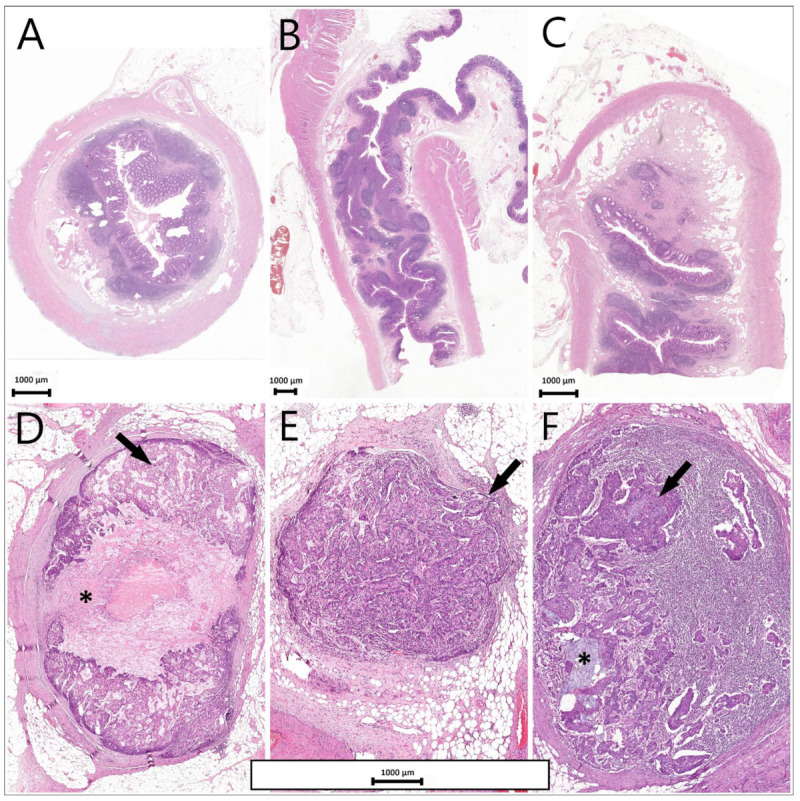
(**A**–**C**). Microscopical examination of the appendix was unremarkable. Hematoxylin and eosin, Full slide scan; (**D**–**F**). Lymph nodes with tumour invasion (arrow) and extracellular mucin present (asterisk)—Hematoxylin and eosin, magnification ×5.

The mitotic index in our case was high (>20/10 high power fields). The tumour showed lymphatic, intra- and extramural venous invasion, perineural invasion and positive circumferential margin. Subserosal tumour deposits were present (pT3). The appendix was microscopically unremarkable (Figure 5A–C). 32 regional lymph nodes were assessed, of which 12 showed tumour invasion (Figure 5D–F). The five colonic polyps identified were diagnosed as tubular adenomas with low-grade dysplasia/intraepithelial neoplasia.

The final diagnosis was high-grade extra-appendiceal goblet-cell adenocarcinoma of the cecum, G3 (high-grade pattern assessed in >50% of the tumour volume).

Differential diagnoses included neuroendocrine tumour (NET), signet ring cell adenocarcinoma and mucinous adenocarcinoma. NET was ruled out by the presence of mucin-exhibiting cells and extracellular mucin; the latter two considered alternatives were excluded by identifying low-grade goblet cell adenocarcinoma component and due to the fact that mucinous cells and poorly cohesive signet-ring-like cells did not exceed >50% of the tumour’s volume.

On postoperative day 4, the general condition of the patient degraded. Low blood pressure, tachycardia, moderate dyspnea and laboratory tests showing increased serum creatinine (4.27 mg/dL) and urea (169 mg/dL) levels determined the patient’s admission in the intensive care unit. Computed tomographic scans showed right pleural effusion, a condensation process of the lower right lung lobe suggestive of pneumonia, bilateral lamellar atelectasis and pulmonary emphysema. The patient died on postoperative day 26 due to pneumonia and acute-on-chronic renal injury.

## 3. Discussion

Because of its unique histological features as a hybrid tumour, GCA has always been a debatable topic among specialists. Most frequently known as goblet cell carcinoid due to its neuroendocrine features, it was first described in 1969 by Gagne et al. [1]. Goblet cell adenocarcinoma was chosen as an accurate denomination to indicate this appendiceal tumour’s amphicrine character in the fifth edition of the WHO classification of tumours of the digestive system [1].

Goblet cell adenocarcinomas have been reported predominantly in the fifth to sixth decade of life, with an estimated incidence of up to 0.05 per 100,000/year and an ethnic preference for Caucasians with no particular gender disparity [1,2,7,8,10,12,13,14,15]. Based on our literature review, eleven cases of extra-appendiceal GCA with the colorectal primary site have been diagnosed so far (Table 3 and Table 4) [1,10,16,17,18,19,20,21].

Patients with extra-appendiceal GCA of the colorectum are most frequently hospitalised for altered general state, weight loss, emesis, anaemia, abdominal discomfort and bowel obstruction [1,2,10,16,17,18,19,20]. Our patient had predominantly anaemia-related symptoms despite the stenosing nature of the tumour. Advanced right colon cancer with increased dimensions frequently generates anaemia due to microscopic or visible haemorrhage and systemic inflammatory response; postoperative complications with longer hospitalisation may occur in such cases [1,22,23]. Pre-operative blood transfusion may generate immunosuppression and cancer recurrence [1,22,24]. A feature that might distinguish GCAs from typical carcinoids is an increase in circulating CEA levels [7,8,13,14,15,24,25]. Our patient exhibited normal CEA levels when hospitalised. Local lymph node metastases are frequent in GCAs [8,13]. Patients with extra-appendiceal GCA frequently present in advanced stages [3,11]. These tumours can spread in the peritoneal cavity, even when lymph nodes are negative; in such cases, peritoneal and ovarian involvement is a recurring finding [8,13,14]. Compared to adenocarcinomas and neuroendocrine neoplasms, hepatic and pulmonary metastases are rare in GCA cases [8,13,14].

GCA is characterised by both neuroendocrine and glandular differentiation occurring in the same cell, and it is believed it originates in the multipotent stem cells of intestinal mucosa’s crypts [1,8,11,13,26,27]. This neoplasia is composed of goblet-like mucinous cells, signet ring-like cells, endocrine cells and Paneth-like cells, predominantly with tubular pattern resembling intestinal crypts and organoid arrangement of neuroendocrine expressing cells [1,11,27]. Low-grade GCAs show tubular architecture, mild nuclear atypia and rare mitoses; high-grade histological essential features include infiltrating single cells, anastomosing tubules, cribriform or solid tumour masses, large groups of goblet-like or signet-like cells, frequent mitoses with atypia, necrosis and desmoplasia [6]. According to these aspects, GCAs are graded as low-grade G1 when tubular/clustered growth is found in >75% of the tumour, intermediate grade G2 when this architecture is assessed between 50% and 75% and high-grade G3 when <25% [9]. A definitive primary extra-appendiceal GCA diagnosis should include the microscopic assessment of the appendix and its exclusion as the tumour’s origin [1]. Immunohistochemical markers such as neuronal specific enolase, synaptophysin, chromogranin A and CD56 are not always positive in GCAs, and tumours with absent neuroendocrine immunoreactivity may exist [7,11,28,29]. Our case highlighted strong and diffuse staining of the tumour cells with synaptophysin and neuronal-specific enolase and negative immunoreaction for chromogranin A. Following microscopic examination and immunohistochemical testing, our diagnosis was high-grade extra-appendiceal goblet-cell adenocarcinoma of the cecum, G3. Although positive neuroendocrine immunostains are not necessary for the diagnosis of GCA, they remain very useful to differentiate it from other tumours like mixed neuroendocrine-non-neuroendocrine neoplasms (MiNENs), mucinous adenocarcinoma and signet-ring cell adenocarcinoma, especially intestinal adenocarcinomas with cohesive signet ring cell component (IACSRCC), which exhibit large aggregates of cohesive signet ring cells [1,11]. Ki-67 index is not useful for grading GCAs [7,30,31].

GCAs generally exhibit different genetic mutations than colorectal adenocarcinoma; the HSD17B8 gene is upregulated in GCA, reflecting the endocrine nature of the tumour being involved in numerous endocrine cancers [11,32]. DSCC1 is more upregulated in GCA than colorectal adenocarcinoma; KCNQ1OT1 and MXRA5 are downregulated in GCA, compared to other colorectal cancers [11,33,34,35]. One study revealed that GCAs showcase mutations of genes involved in the WNT signalling pathway, such as CTNNA1, CTNNB1, TRRAP, NOTCH1 and USP9x [1,36]. Frequent genetic mutations found in colorectal adenocarcinoma are not exhibited by GCA, which are usually negative for BRAF, KRAS and SMAD4 [1,7,32,37]. Such mutations affect the therapeutic conduct because these tumours will not respond to cetuximab and panitumumab [7,38]. Mutations of genes involved in chromatin remodelling, like ARID1A, ARID2, KDM6A, and KMT2, were found in other studies [1,39].

Extra-appendiceal GCA does not benefit from any formal guidelines concerning clinical evaluation and treatment because of its extremely low incidence [7]. It is recommended that the patient’s follow-up after treatment be comparable to that of colorectal cancer, usually over a five-year period [8,14]. The surgical technique of GCA corresponds to habitual oncologic principles concerning margins and lymph node excision [7]. The oncological surgical treatment of appendiceal GCA is R0 curative right hemicolectomy, with resection of the proximal and distal margins and total en-bloc removal of the colonic segment with the mesocolon, which includes the regional lymph nodes [39,40]. Tumours of the cecum require central vascular ligation of ileocolic vessels and the right branches of the middle colic vessels, thus ensuring the complete excision of the mesocolon and the maximum number of lymph nodes recovered [41]. Only low-risk carcinomas (pT1, G1-2, N0, R0) may benefit from endoscopic mucosal resection or laparoscopic segmental resection [42]. Adjuvant treatment is necessary for tumours characterised by serous invasion, pT3 or lymph node invasion due to the increased risk of recurrence [42]. Some authors recommend bilateral adnexectomy in post-menopausal patients [8]. The chemotherapeutic choice is challenging because the management of appendiceal GCAs is based on the guidelines for appendiceal adenocarcinoma due to its aggressive behaviour [7,37]. Appendiceal GCAs benefit from the FOLFOX regimen (leucovorin, 5-fluorouracil and oxaliplatin) with variable response [1,5,7,10,12,20,43]. Peritoneal involvement may be managed with cytoreductive surgery and intraperitoneal chemotherapy [1,44].

GCA’s potential and prognosis are intermediate between neuroendocrine tumours and adenocarcinomas [8,10]. According to one study, GCA has a worse outcome than gastrointestinal low-grade neuroendocrine tumour (NET) and frequently shows vascular and perineural invasion [11]. GCAs are recognised to behave more aggressively, like conventional adenocarcinomas [1]. Histologic grading correlates with the patient’s overall survival, independent of stage [11]. Low-grade GCAs have a survival expectancy between 84 and 204 months, intermediate-grade tumours have an overall survival time of 60–86 months, while high-grade and disseminated tumours are 29–45 months [9,45,46].

The strong point of our case report is the microscopic exclusion of the appendix as the tumour’s origin, sustaining extra-appendiceal GCA as a distinct entity. The extremely rare reporting of such cases makes extensive studies difficult to conduct so that international cooperation may be indicated for research advancement concerning extra-appendiceal GCA.

## 4. Conclusions

Extra-appendiceal neuroendocrine expressing goblet cell adenocarcinoma represents a challenging diagnosis; through our case report, we aimed to raise awareness about possible extra-appendiceal localisations of GCAs by presenting a high-grade goblet-cell adenocarcinoma of the cecum, with positive stains for synaptophysin and neuronal specific enolase and MUC 2 immunoreactive goblet cells.

The appendix was microscopically unremarkable, thus making its exclusion as the origin of the tumour possible. An extra-appendiceal GCA diagnosis should mandatorily imply the exclusion of appendiceal origin for an appropriate classification.

Accurate tumour grading is an important feature to report because of its correlation with the patient’s survival. In this case, due to comorbidities-related death, the patient did not benefit from targeted treatment according to the final histopathological diagnosis.

The extreme rarity of this tumour advocates for international cooperation for advanced research in the form of prospective studies with the clear objective of establishing official guidelines for this type of tumour.

## Figures and Tables

**Table 1:** Laboratory tests were performed upon the patient’s admission.

	Parameter	Measured Value at Hospital Admission	Reference Range	Unit
CBC (Complete Blood Count)				
	RBC	2.97	3.8–5.8	mil/µL
	Hemoglobin (Hb)	5.8	12.6–17.4	g/dL
	Hematocrit (Hct)	20.3	37–51	%
	Mean corpuscular volume (MCV)	68.4	81–103	fL
	Mean corpuscular haemoglobin (MCH)	19.5	27–34	pg/cell
	Mean corpuscular haemoglobin concentration (MCHC)	28.6	31–36	g/dL
	White blood cell count (WBC)	5.33	4–10	mil/µL
	Lymphocytes	23.8	20–55	%
	Monocytes	6.9	≤15	%
	Neutrophils	60.9	45–80	%
	Eosinophils	7.5	≤7	%
	Basophils	0.9	≤2	%
	Platelet count	337	150–450	mil/µL
Blood biochemistry				
	Alanine aminotransferase (ALT)	8	<41	U/L
	Aspartate aminotransferase (AST)	11	<40	U/L
	serum creatinine	1.57	<1.2	mg/dL
	C-reactive protein (CRP)	1.65	<0.5	mg/dL
	Gamma–glutamyltransferase (GGT)	14	<50	U/L
	serum potassium	4.2	3.5–5.1	mmol/L
	serum sodium	137	136–145	mmol/L
	alkali reserve	21	22–29	mEq/lCO_2_
	serum urea	57	<49	mg/dL
	serum glucose	92	60–99	mg/dL
	serum iron	32	59–158	µg/dL
Coagulation screening tests				
	International Normalised Ratio (INR)	1.08	0.8–1.2	-
	Partial Thromboplastin Time (PTT)	36.7	<40	seconds
	Quick time	14.4	11.7–15.3	seconds
	ESR	47	<20	mm/h
Tumor markers				
	CEA	3.8	<5	ng/mL
	PSA	0.585	≤4.5	ng/mL

**Table 2:** Antibodies used for immunohistochemical evaluation.

Antibody (Clone)	Isotype (Clonal Status)	Dilution	External Control (Protocol)	Source	Immunoreaction in Our Case (+/−)
**Synaptophysin** **(SP11)**	Rabbit (monoclonal)	Ready to use	Pancreatic islet cells	Ventana Roche	+ (cytoplasmic staining)
**Neuronal specific enolase** **(ZM24)**	Mouse (polyclonal)	1:100–200	Pancreas, brain	Zeta corporation	+ (cytoplasmic staining)
**Chromogranin A (LK2H10)**	Mouse (monoclonal)	Ready to use	Normal appendix	Ventana Roche	−
**CD56** **(MRQ-42)**	Rabbit (monoclonal)	Ready to use	Pancreatic islet cells; pancreatic endocrine cells	Ventana Roche	−
**CDX-2** **(EPR2764Y)**	Rabbit (monoclonal)	Ready to use	Normal colon; colon adenocarcinoma	Cell Marque	+ (nuclear staining)
**CK20** **(SP33)**	Rabbit (monoclonal)	Ready to use	Colon adenocarcinoma	Ventana Roche	+ (cytoplasmic staining)
**CEA** **(CEA31)**	Mouse (monoclonal)	1:200	Colon mucosa; colon adenocarcinoma	Cell Marque	+ (cytoplasmic and membranous staining)
**MUC2** **(CCP58)**	Mouse (monoclonal)	1:50	Colon	Dako	+ (cytoplasmic staining)
**Ki67** **(MIB-1)**	Mouse (monoclonal)	1:100	Tonsil	Dako	+ (nuclear staining—60%)

**Table 3:** Clinical features of 11 cases of colorectal goblet cell adenocarcinomas reported in the literature.

Case Number	Age/Sex	Presentation	Localisation	Treatment	Outcome
**1 (1974)** [16]	71/F	Rectal bleeding	Rectum	N/A	Free of symptoms at her 4-year follow-up after surgery
**2 (1974)** [16]	60/F	N/A	Rectum	N/A	Asymptomatic at her 6-month follow-up after surgery
**3 (1981)** [17]	32/M	Large bowel obstruction/Ulcerative colitis	Splenic colic flexure	5-fluorouracil; streptozotocin	Peripancreatic nodal enlargement 8 months after undergoing surgery
**4 (2012)** [18]	41/F	Abdominal pain Weight loss	Sigmoid	N/A	No recurrence
**5 (2018)** [19]	79/M	Chronic constipation	Ascending colon	N/A	No recurrence or metastasis at his 17-month follow-up after surgery
**6 (2018)** [20]	N/A	N/A	Cecum	N/A	N/A
**7 (2018)** [20]	N/A	N/A	Cecum	N/A	N/A
**8 (2020)** [10]	57/M	Emesis, lower abdominal cramping and pain	Ascending colon with ileocecal valve involvement	N/A	N/A
**9 (2023)** [1]	60/F	Abdominal pain, weight loss, anaemia	Ascending colon with ileocecal valve involvement	-	Patient died due to septic shock (postoperative day 2).
**10 (2024)** [21]	M (age N/A)	Hematochezia Lower abdominal pain	Rectum	Chemotherapy	N/A
**11 (2024)** [21]	M (age N/A)	Hematochezia Lower abdominal pain	Rectum	Chemotherapy	N/A
**12 (Our case)**	82/M	Fatigue, headache and vertigo (anaemia-related symptoms)	Cecum	-	Patient died due to pneumonia and acute-on-chronic renal injury (postoperative day 26)

N/A—Not Available.

**Table 4:** Pathological features of eleven cases of colorectal goblet cell adenocarcinomas reported in the literature.

Case Number (Year of Publication) [Ref.]	Tumour Size (Greatest Dimension)	Grading	Vascular Invasion (+/−)	Perineural Invasion (+/−)	Lymph Node Metastasis (+/−)	Appendix Status
**1 (1974)** [16]	2.2 cm	N/A	N/A	N/A	N/A	N/A
**2 (1974)** [16]	0.8 cm	N/A	N/A	N/A	N/A	N/A
**3 (1981)** [17]	7 cm	N/A	+	N/A	+	N/A
**4 (2012)** [18]	8 cm	N/A	N/A	N/A	+	N/A
**5 (2018)** [19]	1.7 cm (mass 1), 3.4 cm (mass 2), and 1.5 cm (mass 3)	N/A	+	+	N/A	Appendix unremarkable
**6 (2018)** [20]	N/A	N/A	N/A	N/A	N/A	N/A
**7 (2018)** [20]	N/A	N/A	N/A	N/A	N/A	N/A
**8 (2020)** [10]	4.7 cm	G3	+	+	+	N/A
**9 (2023)** [1]	13.5 cm	G3	+	+	+	Appendix unremarkable
**10 (2024)** [21]	3 cm	G3	+	–	+	Appendix unremarkable
**11 (2024)** [21]	4.5 cm	G2	-	+	+	Appendix unremarkable
**12 (Our case)**	4.5 cm	G3	+	+	+	Appendix unremarkable

N/A—Not Available.

## Data Availability

The original data presented in the study are included in the article, further inquiries can be directed to the corresponding author.
